# Alcohol consumption and prostate cancer incidence and progression: A Mendelian randomisation study

**DOI:** 10.1002/ijc.30436

**Published:** 2016-10-08

**Authors:** Clair Brunner, Neil M. Davies, Richard M. Martin, Rosalind Eeles, Doug Easton, Zsofia Kote‐Jarai, Ali Amin Al Olama, Sara Benlloch, Kenneth Muir, Graham Giles, Fredrik Wiklund, Henrik Gronberg, Christopher A. Haiman, Johanna Schleutker, Børge G. Nordestgaard, Ruth C. Travis, David Neal, Jenny Donovan, Freddie C. Hamdy, Nora Pashayan, Kay‐Tee Khaw, Janet L. Stanford, William J. Blot, Stephen Thibodeau, Christiane Maier, Adam S. Kibel, Cezary Cybulski, Lisa Cannon‐Albright, Hermann Brenner, Jong Park, Radka Kaneva, Jyotsna Batra, Manuel R. Teixeira, Hardev Pandha, the PRACTICAL Consortium,, Luisa Zuccolo

**Affiliations:** ^1^ School of Social and Community MedicineUniversity of BristolBristolUnited Kingdom; ^2^ MRC/University of Bristol Integrative Epidemiology Unit, University of BristolBristolUnited Kingdom; ^3^ The NIHR Bristol Nutrition Biomedical Research UnitUniversity Hospitals Bristol NHS Foundation Trust and the University of BristolBristolUnited Kingdom; ^4^ The Institute of Cancer ResearchLondonSM2 5NGUnited Kingdom; ^5^ Royal Marsden NHS Foundation TrustLondonSW3 6JJUnited Kingdom; ^6^ Strangeways Laboratory, Department of Public Health and Primary CareCentre for Cancer Genetic Epidemiology, University of CambridgeWorts CausewayCambridgeUnited Kingdom; ^7^ Institute of Population Health, University of ManchesterManchesterUnited Kingdom; ^8^ The Cancer Council VictoriaCancer Epidemiology Centre1 Rathdowne StreetCarltonVicAustralia; ^9^ Centre for Molecular, Environmental, Genetic and Analytic EpidemiologyThe University of MelbourneVicAustralia; ^10^ Department of Medical Epidemiology and BiostatisticsKarolinska InstituteStockholmSweden; ^11^ Department of Preventive Medicine, Keck School of MedicineUniversity of Southern California/Norris Comprehensive Cancer CenterLos AngelesCA; ^12^ Department of Medical Biochemistry and GeneticsUniversity of TurkuTurkuFinland; ^13^ Institute of Biomedical Technology/BioMediTech, University of Tampere and FimLab LaboratoriesTampereFinland; ^14^ Department of Clinical BiochemistryHerlev Hospital, Copenhagen University HospitalHerlev Ringvej 75DK‐2730HerlevDenmark; ^15^ Cancer Epidemiology Unit, Nuffield Department of Clinical MedicineUniversity of OxfordOxfordUnited Kingdom; ^16^ Surgical Oncology (Uro‐Oncology: S4)University of Cambridge, Box 279, Addenbrooke's HospitalHills RoadCambridgeUnited Kingdom; ^17^ Cancer Research UK Cambridge Research Institute, Li Ka Shing CentreCambridgeUnited Kingdom; ^18^ Nuffield Department of SurgeryUniversity of OxfordOxfordUnited Kingdom; ^19^ Strangeways Laboratory, Department of OncologyCentre for Cancer Genetic Epidemiology, University of CambridgeWorts CausewayCambridgeUnited Kingdom; ^20^ Department of Applied Health ResearchUniversity College London1‐19 Torrington PlaceLondonWC1E 7HBUnited Kingdom; ^21^ Cambridge Institute of Public Health, University of CambridgeForvie SiteRobinson WayCambridgeCB2 0SRUnited Kingdom; ^22^ Division of Public Health SciencesFred Hutchinson Cancer Research CenterSeattleWA; ^23^ Department of Epidemiology, School of Public HealthUniversity of WashingtonSeattleWA; ^24^ International Epidemiology Institute1455 Research Blvd, Suite 550RockvilleMD; ^25^ Mayo ClinicRochesterMN; ^26^ Department of UrologyUniversity Hospital UlmGermany; ^27^ Institute of Human Genetics University Hospital UlmGermany; ^28^ Brigham and Women's Hospital/Dana‐Farber Cancer Institute45 Francis Street‐ASB II‐3BostonMA; ^29^ Washington UniversitySt LouisMO; ^30^ Department of Genetics and PathologyInternational Hereditary Cancer Center, Pomeranian Medical UniversitySzczecinPoland; ^31^ Division of Genetic Epidemiology, Department of MedicineUniversity of Utah School of Medicine, Salt Lake City, UT; ^32^ Division of Clinical Epidemiology and Aging ResearchGerman Cancer Research Center (DKFZ)HeidelbergGermany; ^33^ Division of Preventive OncologyGerman Cancer Research Center (DKFZ)HeidelbergGermany; ^34^ German Cancer Consortium (DKTK), German Cancer Research Center (DKFZ)Heidelberg, Germany; ^35^ Division of Cancer Prevention and ControlH. Lee Moffitt Cancer Center12902 Magnolia DrTampaFL; ^36^ Department of Medical Chemistry and Biochemistry, Molecular Medicine CenterMedical University Sofia2 Zdrave StSofia1431Bulgaria; ^37^ Australian Prostate Cancer Research Centre‐Qld, Institute of Health and Biomedical Innovation and Schools of Life Science and Public Health, Queensland University of TechnologyBNEAustralia; ^38^ Department of GeneticsPortuguese Oncology Institute, Porto, Portugal and Biomedical Sciences Institute (ICBAS), Porto UniversityPortoPortugal; ^39^ The University of SurreyGuildfordSurreyGU2 7XHUnited Kingdom

**Keywords:** alcohol, prostate cancer, alcohol metabolising genes, Mendelian randomisation

## Abstract

Prostate cancer is the most common cancer in men in developed countries, and is a target for risk reduction strategies. The effects of alcohol consumption on prostate cancer incidence and survival remain unclear, potentially due to methodological limitations of observational studies. In this study, we investigated the associations of genetic variants in alcohol‐metabolising genes with prostate cancer incidence and survival. We analysed data from 23,868 men with prostate cancer and 23,051 controls from 25 studies within the international PRACTICAL Consortium. Study‐specific associations of 68 single nucleotide polymorphisms (SNPs) in 8 alcohol‐metabolising genes (Alcohol Dehydrogenases (ADHs) and Aldehyde Dehydrogenases (ALDHs)) with prostate cancer diagnosis and prostate cancer‐specific mortality, by grade, were assessed using logistic and Cox regression models, respectively. The data across the 25 studies were meta‐analysed using fixed‐effect and random‐effects models. We found little evidence that variants in alcohol metabolising genes were associated with prostate cancer diagnosis. Four variants in two genes exceeded the multiple testing threshold for associations with prostate cancer mortality in fixed‐effect meta‐analyses. SNPs within ALDH1A2 associated with prostate cancer mortality were rs1441817 (fixed effects hazard ratio, HR_fixed_ = 0.78; 95% confidence interval (95%CI):0.66,0.91; *p* values = 0.002); rs12910509, HR_fixed_ = 0.76; 95%CI:0.64,0.91; *p* values = 0.003); and rs8041922 (HR_fixed_ = 0.76; 95%CI:0.64,0.91; *p* values = 0.002). These SNPs were in linkage disequilibrium with each other. In ALDH1B1, rs10973794 (HR_fixed_ = 1.43; 95%CI:1.14,1.79; *p* values = 0.002) was associated with prostate cancer mortality in men with low‐grade prostate cancer. These results suggest that alcohol consumption is unlikely to affect prostate cancer incidence, but it may influence disease progression.

## Abstract 2

**What's new?**

Alcohol may spur prostate cancer progression, though it does not appear to affect incidence, according to new analysis. Variation in genes involved in alcohol metabolism affect how much the body is exposed to carcinogenic metabolites. These authors examined 68 genetic variants in alcohol dehydrogenase (ADH) and aldehyde dehydrogenase (ALDH) genes, seeking a link with prostate cancer risk. While they found no evidence that these variants alter prostate cancer incidence, they did show that SNPs in the ALDH1A2 gene affect prostate cancer mortality. From a public health standpoint, these results suggest reducing alcohol consumption could slow prostate cancer disease progression.

**Abbreviations**
ADH: alcohol dehydrogenase
ALDH: aldehyde dehydrogenase
CI: confidence interval
HR: hazard ratio
LD: linkage disequilibrium;
OR: odds ratio
PRACTICAL: PRostate cancer AssoCiation group to Investigate Cancer‐associated ALterations in the genome
SNP: single nucleotide polymorphism

## Introduction

Prostate cancer is the most common cancer in men in developed countries, with 758,700 new cases diagnosed and 142,000 deaths in 2012.^1^ With increasing uptake of prostate‐specific antigen (PSA) testing and the ageing population, prostate cancer incidence is increasing.^2^ The factors influencing prostate cancer incidence and survival after diagnosis are poorly understood, therefore more evidence is needed.^3^

Alcohol is a carcinogen associated with oropharyngeal, liver, breast, colorectal and oesophageal cancers.^4^ Functional variation in the genes involved in alcohol metabolism result in altered exposure to the carcinogenic metabolites of ethanol, suggesting a mechanism for genetic sensitivity to alcohol to influence the pathogenesis of cancers.^5^ For example, populations with an increased prevalence of common genetic variation in the alcohol dehydrogenase gene, that results in reduced enzyme activity, have an increased risk of oesophageal cancer compared with populations with the fully active enzyme.^4^

At present the role of alcohol use on prostate cancer remains uncertain. The World Cancer Research Fund's extensive report based on systematic reviews described the evidence as limited and inconclusive,^6^ and the International Association for Research on Cancer did not list this cancer site amongst others more apparently caused by alcohol in their Monograph on alcohol's carcinogenicity.^4^ There have been conflicting reports of possible associations of alcohol with various stages or histological grades of prostate cancer,^7^, ^8^, ^9^, ^10^, ^11^, ^12^ and meta‐analyses have highlighted the inconsistencies, emphasising the need for further research in this area.^13^, ^14^

The majority of evidence about the effects of alcohol on prostate cancer is from observational studies. One potential limitation of traditional observational research is that the findings can potentially be explained by common causes of both exposure and outcome (confounding factors). Other potential sources of bias are reverse causation and recall bias, where having prostate cancer affects drinking behaviour or its reporting, rather than alcohol consumption increasing the risk of prostate cancer. A prospective study design could mitigate both of these problems, but could still be affected by bias in the form of the “sick quitter” effect, where former heavy drinkers reduce their alcohol intake in middle‐age because of comorbidities that may be alcohol‐related.

Mendelian randomisation is an approach that uses genetic variants robustly associated with exposures of interest, or their metabolic effects, as instrumental variables to test the un‐confounded and unbiased causal effects of those exposures and their metabolic effects with cancer.^15^ Mendelian randomisation analyses rely on two approximate laws of Mendelian genetics,^15^, ^16^ that at meiosis alleles segregate without any influence of environmental factors and that the inheritance of one trait is independent of the inheritance of others. This allows genetic variation to be used in epidemiological studies as an un‐confounded proxy for an environmental exposure,^15^, ^16^, ^17^ in this case alcohol consumption, to estimate the influence of cumulative life‐time risk of exposure, to reduce recall bias and the “sick‐quitter” effect and to negate reverse causation. All these features are limitations of previous conventional observational studies.^7^, ^8^, ^13^ Mendelian randomisation has already been used successfully in both cardiovascular^18^, ^19^ and cancer epidemiology^20^, ^21^, ^22^, ^23^, ^24^ to clarify the causal effects of alcohol on disease.

In this study, we undertook Mendelian randomisation analyses in which we used variants in alcohol metabolising genes influencing metabolism and intake, to test the causal effect of alcohol exposure on prostate cancer risk and progression. The motivation is that if alcohol intake causally increases prostate cancer risk or progression, then genetic variants associated with metabolic effects of alcohol or increased intake will be differentially represented in cases and controls. We stratified the analysis by histological prostate cancer grade, based on Gleason score, as low‐ and high‐grade prostate cancers have differing natural histories which could be influenced by different risk factors.

## Material and Methods

### Study populations

We used phenotypic and genotypic data from 46,919 men (23,868 cases) in the international Prostate cancer association group to investigate cancer‐associated alterations in the genome (PRACTICAL) consortium. Data were provided by 25 studies within the consortium, based in USA, Australia and European countries. This study population was limited to those of European ethnicity. The studies used a number of methods of recruitment, including screen and clinically detected cases and participants selected due to a family history of prostate cancer. The background characteristics of the participants of each study are shown in Table 1. Gleason scores were used to categorise cancers as low grade (Gleason score ≤6) or high grade (Gleason score ≥7). Further details are available from the consortium website (practical.ccge.medschl.cam.ac.uk). All studies adhered both to national ethical guidelines and to the principles of the Declaration of Helsinki.

**Table 1:** Background information on participants contributing to the PRACTICAL Consortium by study

				**Age at diagnosis**		**PSA level at diagnosis (ng/ml)**			Family history	Gleason score
**Study**	**Country**	**Controls**	**Cases**	**Mean**	**SD**	**Median**	**Lower quartile**	**Upper quartile**	of disease	8–10
CAPS	Sweden	664	1,153	66.10	7.75	13.0	7.0	30.0	17.35%	15.26%
CPCS1	Denmark	2,771	848	69.51	7.91	15.0	8.0	37.5	8.21%	26.65%
CPCS2	Denmark	1,009	265	64.88	6.82	9.0	6.0	14.5	14.72%	9.06%
EPIC	Europe	1,079	722	64.87	5.62	8.6	6.0	15.9	–	2.22%
EPIC‐Norfolk	UK	917	484	72.08	7.56	19.8	19.8	19.8	2.48%	1.86%
ESTHER	Germany	318	313	65.52	5.09	6.9	5.0	14.0	10.54%	8.63%
FHCRC	USA	730	761	59.73	7.18	6.4	4.7	9.8	21.68%	10.38%
IPO‐Porto	Portugal	66	183	59.33	5.23	7.4	5.5	10.1	20.00%	15.85%
MAYO	USA	488	767	65.24	6.42	7.8	4.9	14.7	29.07%	28.42%
MCCS	Australia	1,170	1,698	58.45	8.46	5.4	0.0	11.4	23.45%	10.31%
MEC	USA	829	819	69.53	7.62	–	–	–	13.03%	34.55%
MOFFITT	USA	100	414	64.97	8.27	5.6	4.3	7.4	22.76%	11.11%
PCMUS	Bulgaria	140	151	69.27	8.71	15.8	7.4	34.0	5.30%	29.80%
PPF‐UNIS	UK	188	245	68.86	7.57	8.6	6.3	14.0	25.22%	9.39%
Poland	Poland	359	438	67.66	7.84	11.0	6.9	26.0	10.57%	11.42%
ProMPT	UK	0	166	66.33	8.64	8.8	5.7	15.3	34.62%	16.87%
ProtecT	UK	1,474	1,542	62.76	5.11	5.1	3.8	8.2	7.91%	5.64%
QLD	Australia	87	186	61.32	6.91	5.2	2.2	7.5	36.18%	3.76%
SEARCH	UK	1,244	1,371	63.08	4.76	8.8	5.6	15.0	16.24%	10.14%
STHM1	Sweden	2,224	2,006	66.17	6.99	–	–	–	20.18%	7.93%
TAMPERE	Finland	2,413	2,754	68.18	7.96	8.6	5.6	16.3	–	13.76%
UKGPCS	UK	4,182	4,549	63.76	7.97	9.8	5.6	24.7	23.42%	14.13%
ULM	Germany	354	603	63.78	6.66	9.0	6.0	15.1	44.94%	12.11%
UTAH	USA	245	440	62.57	8.85	–	–	–	51.36%	15.45%
WUGS	USA	0	990	60.80	7.03	5.0	4.0	7.0	42.43%	7.88%

Abbreviations: SD, standard deviation; PSA, prostate‐specific antigen.

### Genotyping data

The participants were genotyped using a custom Illumina Infinium genotyping array (iCOGS), which was specifically designed for the Collaborative Oncological Gene‐environmental Study (COGS) and recorded 211,155 SNPs (details available from: http://ec.europa.eu/research/health/medical-research/cancer/fp7-projects/cogs_en.html).^25^, ^26^ The iCOGS array was designed to investigate SNPs in regions thought to be associated with breast, ovarian and prostate cancer; 68,638 of the SNPs on the array were chosen because of their potential role in prostate cancer aetiology. The other 125,877 SNPs were selected on the basis of potential importance for other cancers and common SNPs known to be associated with any other traits. Individuals with fewer than 95% of genotypes called, or high or low heterozygosity (*p* < 1 × 10^−5^) were dropped from our analysis. In total 201,598 SNPs passed quality control. The genotypic data were used to impute SNPs which were not directly genotyped, but were in linkage disequilibrium (LD) with genotyped SNPs. We used the HapMap 2 CEU reference panel and IMPUTE2 software.^27^

In this study, we searched the iCOGS array database for all alcohol‐metabolising genetic variants (within Alcohol Dehydrogenases (ADHs) or Aldehyde Dehydrogenases (ALDHs) genes), and identified 68 common variants in 5 distinct genomic regions: the ADH cluster on chromosome 4, comprising ADH1A, ADH1B, ADH1C and ADH7; ALDH1A1 and ALDH 1B1 on chromosome 9; ALDH1A2 and ALDH1A3 on chromosome 15. Of these SNPs, 67 were directly genotyped, and one was imputed. An overview of the genes' role in alcohol metabolism and behaviour is shown in Supplementary material Table S1. The characteristics of the SNPs included in this study are shown in Supplementary material Table S2.

### Statistical analysis

We converted the genotypic data for each SNP into a count of the number of minor alleles at each locus. We used logistic regression to estimate the associations of the SNPs with prostate cancer risk (cases *vs*. controls), for all cases and stratified by high‐ *vs*. low‐grade disease. In a case‐only analysis, we used Cox proportional hazards regression to estimate associations of each SNP with prostate cancer‐specific mortality, stratified by grade of prostate cancer. All regression analyses were adjusted for the first 8 principal components of population stratification, since these genomic regions show marked variation across different populations and so do prostate cancer incidence and survival. When checking the proportional hazards assumption, we found little evidence of violation. These regressions were performed for each study and then meta‐analysed using both fixed‐ and random‐effects models. Studies were excluded from the survival meta‐analysis if there were <5 deaths during the follow up period or <90% completion of follow up data. We investigated between‐study heterogeneity using the Stata *metan* command to estimate the *I* ^2^ statistic assuming a fixed‐effect model; we also report random‐effect models for completeness as such models may be relevant where *I* ^2^ values are high (*e.g*., >75%).^28^ Using meta‐regression, we investigated whether heterogeneity could be explained by the following *a priori* defined study‐specific characteristics: mean age at diagnosis, mean PSA at diagnosis, country of study (USA *vs*. elsewhere), and the percentage of participants with a family history of prostate cancer. Manhattan plots of the associations in the five chromosomal regions were constructed to identify SNPs exceeding the Nyholt corrected *p* values threshold for association —a multiple testing correction which accounts for LD between the SNPs.^29^

Sensitivity analyses were conducted by reclassifying low‐ and high‐grade disease as <8 and 8–10 Gleason grade, respectively.

The power of our study was also assessed using reverse power calculations to demonstrate the effect size we would expect to detect given our sample size and *α* = 0.05 with SNPs of a range of minor allele frequencies.^30^ The analysis was carried out using Stata v.13.1. The statistical code used to produce these results can be accessed here (https://github.com/nmdavies/practical-alcohol/).

## Results

The background characteristics of the participants are summarised in Table 1, by study. Variation between studies reflects their individual recruitment methods (*e.g*., some studies selected for those men with a positive family history of prostate cancer).

**Figure 1:**
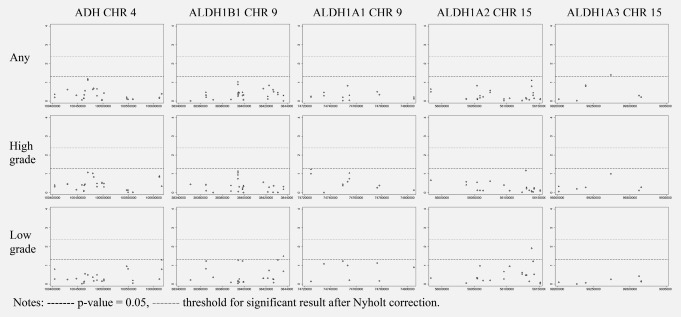
Manhattan plots of association of SNPs, in 5 regions involved in alcohol metabolism, with Prostate Cancer Diagnosis by Prostate Cancer grade.

The Manhattan plots with results from both fixed and random effects meta‐analyses testing associations between SNPs in the five genomic regions and prostate cancer risk are presented in Figure 1 (detailed results available in Supplementary material Tables S4–S9, and sensitivity analyses with alternative definitions of low‐ and high‐grade presented in Supplementary material Tables S16–S19). The figure and Supplementary material tables show that no SNP exceeded the Nyholt corrected *p* values threshold for association with prostate cancer risk.

**Figure 2:**
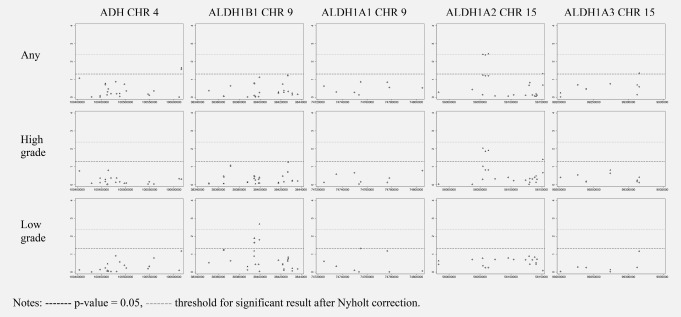
Manhattan plots of association of SNPs, in 5 regions involved in alcohol metabolism, with prostate cancer‐specific survival by prostate Cancer grade.

**Figure 3:**
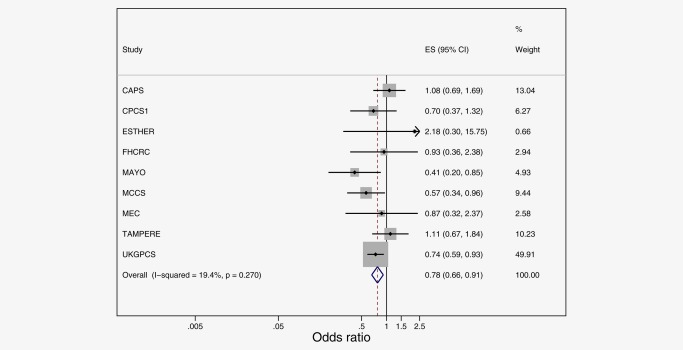
Meta‐analysis of prostate cancer‐specific survival following a diagnosis of any prostate cancer, in association with rs1441817 in ALDH1A2. [Color figure can be viewed at wileyonlinelibrary.com]

**Figure 4:**
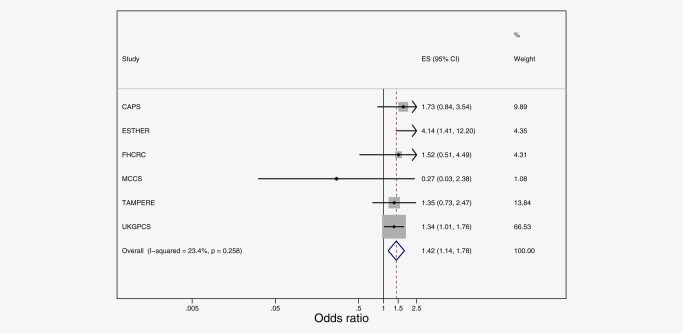
Meta‐analysis of prostate cancer‐specific survival following a diagnosis of low grade prostate cancer, in association with rs10973794 in ALDH1B1. [Color figure can be viewed at wileyonlinelibrary.com]

In case‐only analyses, four SNPs exceeded the Nyholt corrected *p* values threshold for association with prostate cancer‐specific mortality in the fixed‐effect meta‐analysis (summary Manhattan plots presented in Figure 2, and individual SNP results presented in Supplementary material Tables S10–S15, with results of sensitivity analyses with alternative definitions of low‐ and high‐grade presented in Supplementary material Tables S20–S23). Three SNPs within ALDH1A2 were associated with prostate cancer mortality following diagnosis with any prostate cancer: rs1441817 (fixed effects hazard ratio, HR_fixed_ = 0.78; 95% confidence interval (95%CI):0.66,0.91, *p* values = 0.002, *I* ^2^ = 19.4); rs12910509, HR_fixed_ = 0.76; 95%CI:0.64,0.91, *p* values = 0.003, *I* ^2^ = 23.0); and rs8041922 (HR_fixed_ = 0.76; 95%CI:0.64,0.91, *p* values = 0.002, *I* ^2^ = 25.5). To identify the top independent signal amongst these three, we conducted jointly adjusted analyses. Levels of pairwise LD were too high to attempt study‐specific analyses (pairwise LD rs1441817 and rs12910509 *r* ^2^ = 0.89, rs1441817 and rs8041922 *r* ^2^ = 0.88, rs12910509 and rs8041922 *r* ^2^ = 0.99).^31^ Pooled analyses were conducted to estimate the joint effects of rs1441817 and rs12910509/rs8041922 (*r* ^2^ = 0.99) on prostate cancer survival, with a random effect correction for standard errors. These showed an independent effect of rs1441817, similar in size to that of univariate analyses, but no independent effect of rs12910509/rs8041922 once adjusting for rs1441817. Given the high LD between the three SNPs, they should be taken as representing one underlying genetic signal. Figure 3 presents the forest plot of individual studies contributing to the meta‐analysis of prostate cancer‐specific survival in association with the top independent signal in ALDH1A2. A fourth SNP, rs10973794 in ALDH1B1 (also close to IGFBPL1), was associated with prostate cancer mortality in men with a diagnosis of low‐grade prostate cancer (HR_fixed_ = 1.43; 95%CI:1.14,1.79, *p* values = 0.002, *I* ^2^ = 23.4, Fig. 4). This result was robust to changing the low‐grade definition to <8 (HR_fixed_ = 1.23; 95% CI:1.06,1.41, *p* values = 0.002, *I* ^2^ = 0, Supplementary material Table S22).

In general, random effects meta‐analyses yielded weaker evidence of association than fixed‐effect models, as one would expect due to variability across studies producing larger confidence intervals for the former. However, levels of heterogeneity as quantified by the *I* ^2^ statistics were low, with point estimates remarkably similar across the two types of meta‐analysis. Meta‐regression analyses found limited evidence that the study‐level characteristics examined had a strong influence on the pooled results (Table 2).

**Table 2:** Results of univariate meta‐regressions to test if the association of the two SNPs (representing the two signals observed) is affected by selected study characteristics

			Confidence intervals		
Single nucleotide polymorphism	Study characteristic	Ratio of odds‐ratios	**Lower**	**Upper**	*p* Values
rs1441817	PSA	1.48	0.75	2.93	0.19
(ALDH1A2)	FHX	0.63	0.35	1.14	0.10
	USA	0.69	0.30	1.57	0.30
	Age	1.58	1.00	2.51	0.05
rs10973794	PSA	0.65	0.23	1.80	0.33
(ALDH1B1)	FHX	0.65	0.25	1.68	0.27
	USA	1.30	0.29	5.72	0.65
	Age	1.30	0.62	2.72	0.40

PSA, mean PSA at diagnosis.

FHX, percentage family history.

USA, study location in USA *vs*. rest of world.

Age, mean age at diagnosis.

## Discussion

Using data from the PRACTICAL Consortium, we pooled data from 25 studies including a total of 23,868 prostate cancer cases and 23,091 controls, to investigate the association of 68 SNPs within genes thought to be involved with alcohol metabolism with prostate cancer risk and prostate cancer‐specific mortality (amongst men diagnosed with prostate cancer), overall and by Gleason grade. After correcting for multiple testing in the fixed‐effect meta‐analysis, no SNPs exceed the Nyholt threshold for association with a diagnosis of prostate cancer, whereas three SNPs in ALDH1A2 (in strong LD with each other, therefore representing one signal only) exceed the Nyholt threshold for association with prostate cancer‐specific survival. One SNP in ALDH1B1 also exceeds the Nyholt threshold for association with prostate cancer‐specific survival in low‐grade prostate cancer.

### Genetic variants in alcohol metabolising genes

There is evidence that genetic variations in ADH and ALDH genes affecting ethanol metabolism^32^, ^33^, ^34^, ^35^ are associated with altered alcohol intake and risk of alcohol dependence.^31^, ^35^, ^36^, ^37^ The most extensively studied SNP in the context of alcohol intake is rs1229984 in ADH1B. It has been shown to be associated with increased adverse effects from alcohol intake and reduced consumption.^35^, ^36^ On average minor allele carriers drink 17.2% fewer units/week (95%CI:15.6%, 18.9%), are less likely to be in the top third of alcohol drinking volume (odds ratio, OR = 0.7; 95%CI:0.68,0.73) and are less likely to binge drink (OR = 0.78; 95%CI:0.73,0.84).^23^ It has also been reported to affect cancer risk at various sites.^5^ In our study, the associations of this SNP with prostate cancer diagnosis and survival were OR_fixed_ = 1.00 (95%CI:0.96,1.03, *p* values = 0.87), and HR_fixed_ = 1.11 (95%CI:0.95,1.30, *p* values = 0.17), respectively. Combining the effects of the ADH1B SNP on alcohol intake with the upper confidence intervals from our results implies that a 17% reduction in alcohol consumption is unlikely to reduce prostate cancer risk by >3% and prostate cancer mortality by >5%.

Alcohol is metabolised to acetaldehyde, a known carcinogen, and there is evidence to support the theory that genetic variants in alcohol metabolising genes, which control the production and breakdown of acetaldehyde, contribute to carcinogenesis.^4^, ^5^, ^20^, ^24^ There is also evidence of a tissue‐specific interaction in the prostate between ethanol and retinoic acid, through modulations of ALDH1A1, ALDH1A2 and ALDH1A3 levels.^38^ To our knowledge, this is the first comprehensive investigation of the association between ADH and ALDH variants, as genetic proxies for alcohol, and prostate cancer to date. Genetic predisposition to prostate cancer has been examined by GWASs, which shows common genetic variants can explain 33% heritability of prostate cancer but no genome‐wide significant hits are in ADHs or ALDHs^39^, ^40^ (however, this lack of evidence from GWASs could be a type 2 error). Similarly, we did not find any evidence of genetic association between ADH/ALDH variants and prostate cancer incidence in this study. Possible reasons for this include type 2 error, especially if the underlying effects of alcohol on prostate cancer incidence are small and limited to the very heavy drinking behaviours and/or to the more aggressive forms of disease, as possibly suggested by the recent literature.^7^, ^12^

We have shown that SNPs in ALDH1A2 are associated with altered prostate cancer‐specific mortality in a case‐only analysis. None of these SNPs appear to have regulatory features (www.ensemble.org), so they are unlikely to be causal variants themselves but rather they could be in LD with the causal variants. Recently, ALDH isoforms have been suggested as possible mechanistic mediators of metastasis in prostate cancer in particular^41^ and other solid tumours in general.^42^ One study found lack of compelling evidence linking variation in ALDH1 (including ALDH1A1, ALDH1A2, ALDH1A3 and ALDH1B1) with prostate cancer progression,^41^ but another had reported preliminary evidence for a potential role of ALDH1A2 as a tumour suppressor gene in prostate cancer cell lines^43^ and decreased expression of ALDH1A2 has been associated with shorter recurrence free survival in patients with prostate cancer.^43^ In our study, three intronic SNPs in ALDH1A2 were associated with longer survival, none of which were directly or indirectly (through LD) associated with alcohol‐related phenotypes (http://www.ebi.ac.uk/gwas/). One potential explanation for our results may be that these SNPs, or others in LD with them, lead to increased activity in ALDH1A2. We speculate that the observed prostate cancer survival effect could be the result of a net increase in the synthesis of retinoic acid (by ALDH1A2), which is particularly beneficial when the rate of conversion is affected by slower ADH activity in the presence of alcohol consumption (retinol and ethanol both being ADH substrates,^44^ and ethanol modulating retinoic acid synthesis in the rat prostate^38^).

Another intronic SNP in ALDH1B1 (also close to IGFBPL1) was found to be associated with increased mortality following a diagnosis of low‐grade prostate cancer. ALDH1B1 is the second most abundant mitochondrial ALDH, after ALDH2, with documented involvement in alcohol metabolism and dependence.^45^ However, this specific SNP is not known to be in LD with any of the variants associated with alcohol phenotypes to date, therefore we cannot speculate on its specific role in relation to alcohol. Evidence has been previously found linking levels of ALDH1B1 to survival following gastric cancer^46^ and non‐small‐cell lung cancer,^47^ but not prostate cancer,^41^ however the latter was a study *in vitro* Conversely,ALDH1A1 expression in the prostate has been reported to be a good candidate prognostic biomarker, based on all cause mortality and to a lesser extent prostate cancer‐specific mortality,^48^ and ALDH1A3 expression is thought to be involved with initiation and progression of several cancers,^49^ however we did not observe an association with common germ‐line mutations in either of these genes, or did we observe associations with variation in ADH genes. This could be due to different functional effects of variants on metabolic levels (*e.g*., alcohol and acetaldehyde peak levels and cumulative concentrations), affecting prostate cancer proliferation or survival differently both in terms of effect sizes and pathways.

### Differences by tumour grade

In this study, we have found that genetic variants in genes involved in alcohol metabolism were associated with disease‐specific mortality in men with prostate cancer, most of whom had been diagnosed with low‐grade disease. We found a signal specific to low‐grade prostate cancer survival, but none for high‐grade disease. Possible reasons why stronger associations were not seen with high‐grade cancers include: limited power, as there were smaller numbers of high‐ compared with low‐grade cases (this was investigated using a reverse power calculation, to assess the power of this study to detect small associations (Supplementary material Table S3)); patients behaviour may change following a diagnosis, *e.g*. patients with high‐grade prostate cancer may be too ill to drink and the effect of the SNPs cannot be seen in the absence of drinking; or the findings could reflect a true clinical difference in the way alcohol affects survival for the different grades of prostate cancer.

### Strengths and limitations

The strengths of this study include the large sample size and availability of data on both risk and mortality stratified by grade, which is an important predictor of prognosis. Importantly, we used a Mendelian randomisation approach, which minimises the potential for bias due to confounding, information bias (recall bias and sick‐quitter effect) and reverse causation, major limitations of previous studies in this area.^7^, ^8^, ^13^ We were also able to control for confounding by population stratification by adjusting for basic population characteristics. A potential limitation of our study is its power to detect small effects of alcohol on high‐grade disease, with fewer of these cases having been diagnosed and followed‐up.

Data for this study were contributed to the PRACTICAL Consortium from many studies with varying recruitment and inclusion/exclusion criteria, as different screening practices could complicate the interpretation of our results.^50^ For example, CAPS participants were all diagnosed clinically, whereas ProtecT participants were all screen detected. While the consortium provides a large sample size for investigation, there is inevitably some heterogeneity in the contributing studies. The effect of this was investigated using random effects meta‐analysis and meta‐regression. No one study‐level characteristic had a strong influence on the results, and we were unable to clearly determine the reason for the modest levels of heterogeneity observed, and the consequent variation between the fixed‐effect and random‐effect analyses. Potential explanations include: systematic differences in smaller *vs*. larger studies, and the former being assigned larger weights in random‐effect models; true variation in the effects of alcohol in the different study populations; effects of study designs that we were not able to investigate, *e.g*. the different ways cases were ascertained/recruited.

Another possible limitation to consider is the potential influence of pleiotropy. There may be other direct pathways through which the SNPs influence prostate cancer mortality independently of alcohol metabolism and intake. In particular, SNPs in ALDH1A2 could have a role in retinoic acid synthesis, which could affect cancer survival *per se* and in conjunction with alcohol,^38^ and we note that the ALDH1B1 SNP is in close proximity to IGFBPL1, which may encode a putative tumour suppressor protein.^51^ However, there were no other associations of these SNPs, or others in LD with them, reported by the catalogue of published genome‐wide association studies (http://www.ebi.ac.uk/gwas/); therefore the risk of pleiotropy for the genetic variants under study here is likely to be small. Finally, the direction of effect of the SNPs on alcohol intake, apart from rs1229984, is unknown so it is not possible to estimate the effect size of the four SNPs we found to be associated with survival.

### Future directions

There are a number of ways in which this work could be taken forward. These include further analysis in larger consortia with longer follow up data availability, or repeating within individual subsets with certain study design characteristics to increase similarity of studies included in analysis (direct replication). Analysis of further genetic variants with known effects on alcohol metabolism or behaviour would also allow further development of this work (indirect replication), as would establishing the magnitude and direction of effect of genetic variants in alcohol metabolising genes on alcohol intake. It would also be interesting to investigate patient behaviour following diagnosis to establish if the varied effect seen between high‐ and low‐grade disease could be due to differences in behaviour.

## Conclusion

If confirmed in independent studies or through direct or indirect replication, these findings suggest a role for alcohol in the progression of prostate cancer, whilst also confirming that alcohol is unlikely to have a large impact on prostate cancer carcinogenesis. This has potential public health implications and alcohol intake could be targeted to improve survival from prostate cancer as part of holistic care.
